# Open education in eye health: transforming access to learning

**Published:** 2018-02-08

**Authors:** Daksha Patel, Sally Parsley, Astrid Leck

**Affiliations:** 1 E-learning Director: International Centre for Eye Health, London School of Hygiene and Tropical Medicine, London, UK.; 2 E-communications manager: International Centre for Eye Health, London School of Hygiene and Tropical Medicine, London, UK.; 3 Research fellow: International Centre for Eye Health, London School of Hygiene and Tropical Medicine, London, UK.

**Remaining relevant and keeping up with medical advances is a challenge, as access to high-quality education is inequitable and costly. The open education approach is designed to reduce restrictions to learning.**

**Figure:**
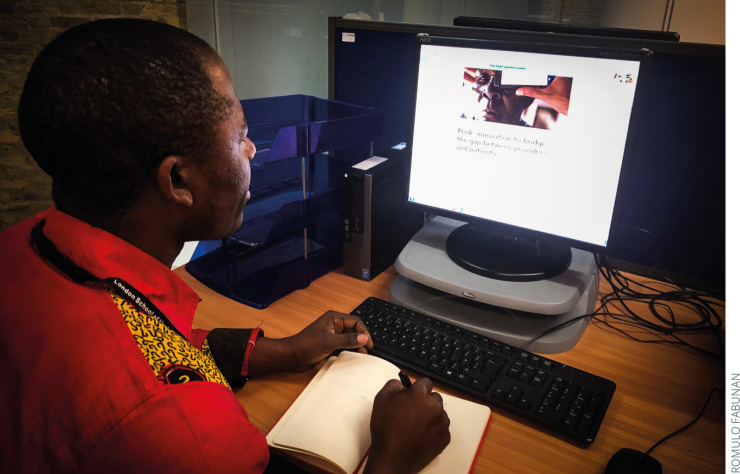
Programme manager and outreach coordinator Mathew Mbwogge (Cameroon) uses ICEH's Global Blindness course, which is available free of charge on FutureLearn, to update his knowledge. UK

Over the years, improvements in medical education have been linked with improved clinical practice, which has in turn contributed to a doubling of life span in the 20th century.^1^ However, we are still faced with inequities in health provision within and between countries. Health systems worldwide continue to place growing demands on health professionals to keep up with medical advances, manage challenges due to rapid demographic change and remain prepared to face new infections or environmental risks.

In eye health education, the situation is no different. Both practitioners and training programmes must review and update their resources to remain relevant by:

- Setting standards for clinical and surgical competencies
- Ensuring that training programmes are matched to the needs of the population, in the context of their health systems
- Improving teamwork across professional groups, such as ophthalmologists and optometrists.

**Inequalities**

Inequalities in health exist for various reasons. If inequalities are avoidable (e.g. by making eye services more affordable, or making them available in rural areas) then they are better described as inequities, a word which highlights the unfairness of the situation.

Maintaining high standards for the quality of education in eye health is essential; these should be consistent from country to country. In theory, quality requirements for education in eye health can be aligned by creating international training curricula, practice guidelines and standardised competencies. In practice, however, it is difficult to standardise training due to cost, inequitable opportunities, a shortage of educators and limited access to updated resources. Inequities in education and training can result in low workforce motivation, low clinical and surgical outputs, poor quality of care and dissatisfied patients. In order to address this, medical education needs to keep pace not only with advancements in medicine but also with new educational methods and technologies.

**Open education**

The two most important aspects of openness have to do with free availability over the internet and as few restrictions as possible on the use of the resource. There should be no technical barriers (undisclosed source code), no price barriers (subscriptions, licensing fees, pay-per-view fees) and as few legal permission barriers as possible (copyright and licensing restrictions) for the end-user.

Our everyday life has been transformed by the use of technology to connect with each other, gain access to information and conduct financial transactions. This offers an opportunity to broaden our approach to education and remain relevant to how learners' needs, behaviour and access to information are changing in the digital era. There is growing evidence that online learning (E-learning) can support knowledge and skill development and can also be used to support and strengthen training programmes.^2^

Continuing professional development requires that individuals adopt a purposeful, self-directed approach to find resources that are appropriate to them at that time. E-learning, together with guidance from professional societies, can support eye health workers and professionals to do so.

Open education is about broadening access to learning by removing barriers such as cost and distance. It is usually done by offering access to free open educational resources via the internet. Learning can be guided or self-directed.

The term ‘open educational resources’ first came into use in 2002, when participants at a UNESCO conference defined it as: “The open provision of educational resources, enabled by information and communication technologies, for consultation, use and adaptation by a community of users for non-commercial purposes.”^3^ Not only can users of open educational resources use or read it, but they can also adapt it, build on it and reuse it.

**Figure 1:**
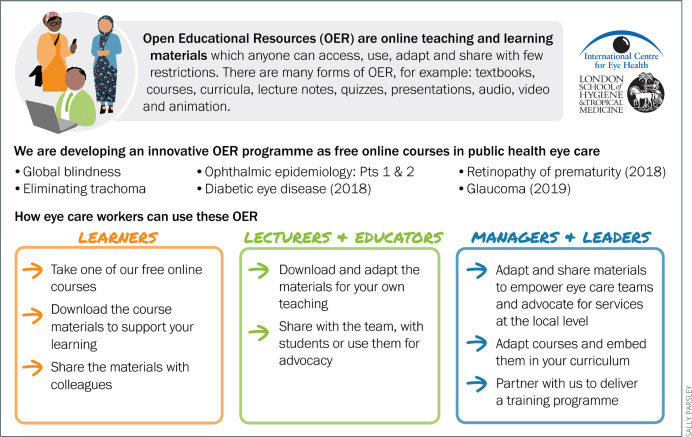
Different ways to use open education In eye health

Open does not mean totally unrestricted; rather it is guided by the Creative Commons licensing framework.^4^ The key principle of this framework is that resources must be attributed to the original creator or source. It also states exactly how content may be used and adapted, provided that it has been attributed correctly:

1. **Retain.** The right to make, own and control copies of the content (e.g., download, duplicate, store, and manage)
2. **Reuse.** The right to use the content in a wide range of ways (e.g., in a class, in a study group, on a website, in a video)
3. **Revise.** The right to adapt, adjust, modify, or alter the content itself (e.g., translate the content into another language)
4. **Remix.** The right to combine the original or revised content with other material to create something new (e.g., incorporate the content into a mashup)
5. **Redistribute.** The right to share copies of the original content, your revisions and/or your remixes.

## Open online education: our approach

In 2015, the International Centre for Eye Health (ICEH) started to develop open education courses in eye health, with the idea of exploring fresh opportunities in technology and addressing imbalances in opportunities for eye health education.

Each course was facilitated over several weeks and learners were able to participate actively in the online discussions. All of the course content was then made available as a range of open educational resources for online use, including videos, images and online articles.

The course structure allowed us to align all these resources into a learning framework (curriculum) with clearly structured learning outcomes and self-assessment tests. These became free-to-access, standalone courses on online learning platforms such as FutureLearn or Moodle.

The purpose behind this approach is to enable individuals to engage easily with the content, download and reuse it locally; and to adapt and introduce it into their own training programmes.

When courses are ‘live’ (facilitated), there are peer discussion forums, webinars with experts who answer learners' questions, and course mentors who guide a learner through the content. This helps to enhance learning and reflects how people share information in a conversational style, as adopted across many social media technologies such Facebook, WhatsApp or Twitter.

Finally, when a user completes the course requirements, they have the option to purchase a certificate as an acknowledgement of their achievement.

## What are the available courses?

ICEH have made a range of courses available:

- **Global blindness: planning and managing eyecare services.** This course is structured to help the learner to understand the WHO classification of visual impairment, the magnitude and causes of blindness and the strategies and planning required to control cataract blindness and refractive errors. There are tools to support learners to begin to apply their learning at a local level.
  *“This course is excellent in explaining how to set aims and objectives and the relevance of national and local planning for eye care.” **Denise***
- **Ophthalmic Epidemiology 1: Basic principles and 2: Application to eye disease.** This is a specific subject area delivered as a two-part course structured to build an understanding of the basic principles of epidemiology as applied to ophthalmology. Participants are guided to critically appraise evidence essential to improve their practice. Quote from a previous participant:
  *“This course made complex materials easy to understand and I could do it in my own time”*
- **Eliminating Trachoma:** Trachoma remains endemic and a risk of blindness in 42 countries. This course is a practical induction into SAFE activities that need to be implemented towards the goal of eliminating trachoma.
  *“I have been able to identify the causative agents, how to diagnose trachoma and strategic plans to eradicate trachoma. Global mapping cannot be over emphasised as it is the only way for us to identify or pinpoint areas that are trachoma endemic.” **Rotimi***
  *“The course has given me a new perspective on trachoma. I think it should be recommended for all ophthalmologists, especially in areas at risk. I've learned a lot about mapping trachoma and I'm eager to learn even more.” **Eloisa***

## Different ways to use open education in eye health

Open education promotes a sense of shared responsibility to ensure that good quality information, of relevance for practice at a local level as well as at the global level, is available. Strengthening an interconnected approach in the digital age has the potential to address inequity in eye health knowledge and encourage the practice of life-long learning for all health professionals.

**Cataract training using simulation**

**Figure:**
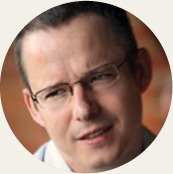
Will Dean

Clinical Research Fellow, London School of Hygiene & Tropical Medicine, London, UK.

Over the last 30 years, there have been significant changes in the way eye surgery is taught to trainee surgeons. A fairly recent development is that, instead of learning eye surgery in a live operating theatre, surgeons can practise surgical techniques by simulation: using another object with properties that are similar enough to those of a real human eye. The idea is to help eye surgeons to learn a new technique, such as cataract or glaucoma surgery, safely and effectively. Surgeons are expected to achieve a specified level of competence and confidence before they carry out supervised surgery on real patients.

Cataract training by simulation can be done using high-tech computerised simulators, for example the HelpMeSee simulator for small-incision cataract surgery (New York, USA), or the Eyesi Surgical Training simulator for phacoemulsification (VRmagic, Mannheim, Germany). However, these may be unaffordable in low- and middle-income countries.

Fortunately, low-cost simulation models are also available. For example, a tomato placed into boiled water for 30 seconds can be used to practise capsulotomy, an apple can be used to simulate scleral tunnelling and a banana or piece of foam is very good for practising suturing. The simulation does not need to be ‘high-tech’ or expensive, but it does need to have high fidelity – i.e., it should be very similar to the real thing. The website **www.simulatedocularsurgery.com** has a simulation gallery with interesting ideas from around the world.

**Figure:**
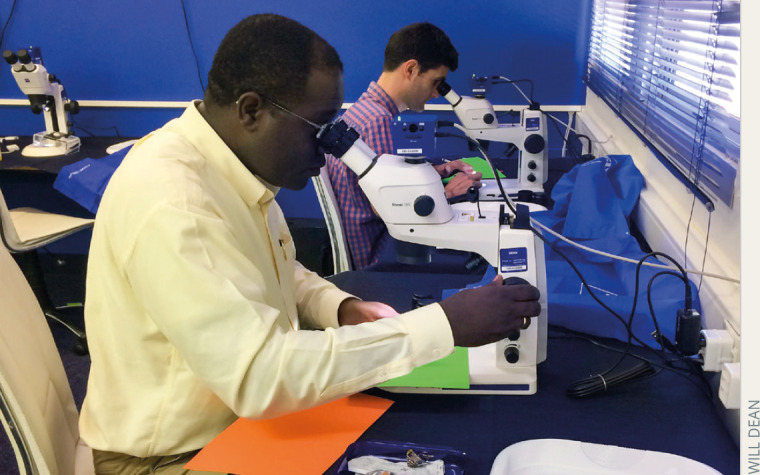
Surgeons practise techniques again and again. SOUTH AFRICA

Simulation can be a powerful tool that allows sustained, deliberate practising of individual surgical steps again, and again, and again. Imagine the benefits of being able to practise a scleral tunnel incision or a capsulotomy 50 to 100 hundred times before operating (under supervision) on a patient. Simulation can help trainee surgeons to practise managing complications during surgery and experienced surgeons to learn new techniques.

Practice makes perfect, but the correct technique must be practised. Simulation training must be integrated into a curriculum. Trained surgical instructors must be present to offer instruction, guidance and feedback. Outcome measurements and assessments will help to ensure the quality of the training.

The International Agency for the Prevention of Blindness (IAPB) have developed a Standard List for the initial development of a surgical skills centre (or wet/dry-lab): **https://iapb.standardlist.org/knowledge/guide-establishing-surgical-skills-centre/**

Although it takes years of experience and practice in the operating theatre to become an expert cataract surgeon, learning cataract surgery to an acceptable level of competence can be achieved safely and efficiently thanks to cataract training by simulation.
